# Synthetic role of miR‐200b‐3p, ABCD^2^ score, and carotid ultrasound in the prediction of cerebral infarction in patients with transient ischemic attack

**DOI:** 10.1002/brb3.2518

**Published:** 2022-03-09

**Authors:** Jiwei Zheng, Zhirong Wang, Na Li, Xiaomeng Zhang, Xiaoguang Huo

**Affiliations:** ^1^ Ultrasound Department Huantai People's Hospital Zibo Shandong China; ^2^ Neurology Department Huantai People's Hospital Zibo Shandong China; ^3^ Ultrasound Department Zibo Central Hospital Zibo Shandong China

**Keywords:** ABCD^2^ score, carotid stenosis, cerebral infarction, miR‐200b‐3p, prediction, transient ischemic attack, ultrasound

## Abstract

**Background:**

Transient ischemic attack (TIA) is a major risk factor for the occurrence of cerebral infarction (CI). This study aimed to evaluate the predictive value of the synthetic role of miR‐200b‐3p, ABCD^2^ score, and carotid ultrasound for CI onset in patients with TIA.

**Methods:**

Expression of miR‐200b‐3p was detected by reverse transcription quantitative PCR and carotid stenosis degree was evaluated using carotid ultrasound examination. Association of miR‐200b‐3p with ABCD^2^ scores and carotid stenosis degree was assessed using *t*‐test and chi‐square test. Logistic regression analysis was used to judge the ability of miR‐200b‐3p, ABCD^2^ score, and carotid ultrasound to predict the occurrence of CI. Receiver operating characteristic curve was used to analyze the diagnostic value of miR‐200b‐3p and the accuracy of miR‐200b‐3p, ABCD^2^ score, and carotid ultrasound in predicting CI development.

**Results:**

Expression of serum miR‐200b‐3p was significantly increased in TIA patients compared with healthy controls, and had diagnostic value in TIA patients. Serum miR‐200b‐3p was significantly associated with dyslipidemia, ABCD^2^ score, and carotid stenosis degree in TIA patients. ABCD^2^ score, carotid stenosis degree, and serum miR‐200b‐3p were independently associated with CI onset, and the synthetic role of these three indicators had the best accuracy in the prediction of CI onset in TIA patients.

**Conclusion:**

Serum miR‐200b‐3p expression was increased in TIA patients with considerable diagnostic value to screen TIA cases from healthy controls. Moreover, we speculated that the combination of miR‐200b‐3p, ABCD^2^ score, and carotid stenosis degree by ultrasound may propose as an efficient predictive strategy for the prediction of CI in TIA patients.

## Graphical abstract

The synthetic role of miR‐200b‐3p, ABCD2 score, and carotid stenosis degree may propose as a potential predictive strategy in the prediction of CI in TIA patients.

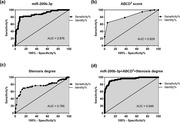

## 1 INTRODUCTION

Transient ischemic attack (TIA) is a sudden, transient neurological dysfunction resulting from a transient inadequate blood supply to focal brain, spinal cord, or retina, causing retinal ischemia (Coutts, 2017; Easton et al., 2009; Hu et al., 2019). Cerebral infarction (CI), also known as cerebral ischemic stroke, is a refractory disease that seriously endangers human health, which is characterized by high morbidity, disability, and mortality (Liang et al., 2018). Although the clinical symptoms of TIA could be completely recovered, 10%–20% of patients would develop CI at an early stage after TIA onset, leading to greater physical damage in patients (Zhang et al., 2020). TIA is considered a major risk factor and an early warning sign that leads to the occurrence of CI (Wang et al., 2020; Zhang et al., 2020). Therefore, accurate diagnosis of TIA and early screening of TIA cases at high risk of CI are of great importance for effective prevention and treatment of CI.

Carotid ultrasound is the cornerstone of screening and diagnosis of carotid artery disease and cerebrovascular disease (Rafailidis et al., 2017). Carotid ultrasound is considered the most important technique to diagnose carotid stenosis (Eckstein et al., 2013). Studies indicated that artery stenosis caused by carotid atherosclerosis was one of the major causes of TIA occurrence (Longstreth et al., 2018). Therefore, to determine the degree of carotid stenosis using carotid ultrasound had a considerable reference value for predicting CI. Furthermore, ABCD^2^ score has also been proposed as a useful tool to predict the occurrence of short‐term CI after TIA onset (Johnston et al., 2007). Until recently, the ABCD^2^ score was the most widely used tool in predicting the occurrence of CI in clinical TIA patients (Cutting et al., 2016; Giles et al., 2017); however, its sensitivity and accuracy are still suboptimal in the short term of TIA (Khorvash et al., 2018). Thus, the methods to predict the risk of CI in TIA patients still need improvement.

MicroRNA‐200b‐3p (miR‐200b‐3p), as one of the highly stable noncoding RNAs, has been implicated in the development and progression of several cerebrovascular‐related diseases (Choi et al., 2011; Kim et al., 2016; Wu et al., 2021). First, in terms of lipid metabolism, Wu et al. (2021) unmasked that silencing miR‐200b‐3p could significantly improve lipid accumulation and cholesterol efflux phenomenon in macrophages. Second, in the process of angiogenesis, early studies reported that miR‐200b‐3p regulated the activity of vascular endothelial cells and angiogenesis and other processes via regulating endothelial growth factor signaling pathways (Choi et al., 2011). Subsequent studies successively demonstrated that miR‐200b‐3p, as an atherosclerosis‐associated miRNA, aggravated atherosclerosis by promoting endothelial cell apoptosis (Kim et al., 2015; Zhang et al., 2021). Moreover, aberrant expression of miR‐200b‐3p was found in tissues of degenerative aortic stenosis patients, and the expression levels were significantly upregulated (Shi et al., 2016). Furthermore, a gene polymorphism study by Kim et al. (2016) pointed out that miR‐200b‐3p was significantly associated with stroke susceptibility and mortality. All the above studies illustrated the significance of miR‐200b‐3p in the occurrence and development of cerebrovascular related diseases. However, neither the expression of serum miR‐200b‐3p in TIA patients nor the possibility of a relationship of miR‐200b‐3p with the occurrence of CI secondary to TIA has been established.

Therefore, by analyzing the data from TIA patients, this study aimed to analyze the expression changes of serum miR‐200b‐3p and its clinical value in TIA patients. The diagnostic potential of serum miR‐200b‐3p to distinguish TIA patients from healthy controls was evaluated, and the predictive value of miR‐200b‐3p combined with ABCD^2^ score and carotid ultrasound detection data was also investigated. The results are expected to provide evidence for a novel predictive strategy to predict CI onset in TIA patients, and thereby to reduce the occurrence of CI.

## 2 MATERIALS AND METHODS

### 2.1 Patients and collection of tissues

According to the TIA diagnostic criteria proposed by American Heart Association/American Stroke Association (Easton et al., 2009), 189 TIA patients admitted to Huantai People's Hospital from 2016 to 2019 were included in this study as subjects. Specific inclusion criteria were as follows: (i) sudden onset of localized neurological deficits with complete resolution of symptoms and signs within 24 h; (ii) patients with clinical manifestations resembling TIA episodes were excluded, such as patients with migraine, epilepsy, peripheral vertigo, periodic paralysis, intracranial aneurysm, atrial fibrillation and hypoglycemia; (iii) no severe chronic history of heart, brain, lung and other important organs; (iv) nonpregnant or lactating. In addition, 106 healthy controls who were free from arterial stenosis and with no history of cardiovascular, cerebrovascular, metabolic, or malignant disease were included in this study. Venous blood samples were collected from TIA patients and healthy controls, and serum samples were collected by centrifugation at 4℃ for subsequent analysis. Collection of blood samples from TIA patients was accomplished within 24 h of onset. Patients were followed for 30 days after TIA onset, and CI occurrence was recorded. CI occurrence was determined based on clinical manifestations of patients and confirmed by the magnetic resonance imaging (MRI) and other relevant tests (Kernan et al., 2014). The patients were followed up till the onset of CI or the end of the 30‐day follow‐up. All protocols for blood sample collection and analysis were followed in accordance with the guidelines of the Ethics Committee and approved by the Ethics Committee of Huantai People's Hospital. A signed informed consent has been obtained from all participants before enrolling them in this study.

### 2.2 Carotid ultrasound examination

All the participants underwent the carotid ultrasound examination, and the artery stenosis degree of bilateral, common and internal carotid arteries were detected by Aplio300 Doppler ultrasound instrument (Toshiba, Inc.) supplemented with a 10 MHz line array probe. The degree of stenosis artery was measured at the narrowest part of the lumen, expressed as a percentage. According to the artery stenosis degree defined by the North American Symptomatic Carotid Endarterectomy Trial method (Paciaroni et al., 1999), the specific grading was as follows: carotid artery stenosis (CAS) grade < 50% was defined as mild stenosis; CAS grade < 70% was defined as moderate stenosis; and CAS grade ≥ 70% was defined as severe stenosis.

### 2.3 ABCD^2^ score

Based on the already published report (Johnston et al., 2007), the ABCD^2^ score criteria were as follows: (i) age ≥ 60 years, taking 1 score; (ii) systolic blood pressure ≥ 140 mmHg or diastolic blood pressure ≥ 90 mm Hg at presentation, taking 1 score; (iii) clinical presentation with unilateral limb weakness, taking 2 scores; language impairment, without limb weakness, taking 1 score; (iv) symptom duration ≥ 60 min, taking 2 scores; 10–50 min, taking 1 score; <10 min, taking 0 score; and (v) with diabetes, taking 1 score. The scores are summed for a total score of 0–7 scores. The grade of risk to develop stroke was graded as: low‐risk group (0–3 scores); medium‐risk group (4–5 scores); and high‐risk group (6–7 scores).

### 2.4 RNA extraction and reverse transcription quantitative PCR

All protocols followed the manufacturer's guidelines in the present study. Total RNA in samples was extracted by TRIzol reagent (Invitrogen, Life Technologies, Paisley, UK). The extracted RNA was reversed transcribed into single‐stranded cDNA with PrimeScript reverse transcriptase kit (TaKaRa, Shiga, Japan). The expression level of serum miR‐200b‐3p was detected by reverse transcription quantitative PCR (RT‐qPCR) with the SYBR green I Master Mix kit (Invitrogen, Carlsbad, CA, USA) on a 7500 real‐time polymerase chain reaction system (Applied Biosystems, USA). U6 was used as an endogenous control for miR‐200b‐3p. The 2^−ΔΔCt^ method was used to compute the expression value of miR‐200b‐3p.

### 2.5 Statistical analysis

The statistical data were expressed as mean ± standard deviation (SD). SPSS 21.0 (SPSS, Inc., Chicago, IL, USA) and GraphPad 7.0 (GraphPad Software, Inc., USA) were used to analyze all statistical data. Kolmogorov–Smirnov test was used to determine the normality of data distribution. Student's *t*‐test and chi‐square test were used to compare the differences between two groups, including the clinical characteristics of the study population. The association of miR‐200b‐3p with ABCD^2^ scoring and carotid stenosis degree was analyzed using *t*‐test and chi‐square test. Logistic regression analysis was used to judge the ability of miR‐200b‐3p, ABCD^2^ scoring, and carotid ultrasound results to predict the occurrence of CI. Receiver operating characteristic (ROC) curve was used to analyze the diagnostic value of miR‐200b‐3p in distinguishing TIA patients from healthy controls, and to evaluate the accuracy of miR‐200b‐3p, ABCD^2^ score, and carotid ultrasound results in predicting cerebrovascular events. Note that *p* < 0.05 was considered to indicate a statistically significant difference.

## 3 RESULTS

### 3.1 Clinical characteristics of the study population

All clinical characteristics of the study population are included in Table 1. Clinical characteristics revealed that there were no significant differences in age, gender, body mass index (BMI), history of hypertension, diabetes, and dyslipidemia between TIA patients (*n* = 189) and healthy controls (*n* = 106) (all *p* > 0.05). In addition, the average ABCD^2^ score of TIA patients was 4.78 ± 1.59; the risk to develop a stroke was based on this score. According to this score, 29 patients (15.34%) were at low risk, 96 patients were (50.80%) at medium risk, and 64 patients (33.86%) were at high risk. For the carotid stenosis degree by ultrasound detection, 12 patients (6.35%) were diagnosed with mild stenosis, 59 patients (31.22%) were with moderate stenosis, and 118 patients (62.43%) were with severe stenosis.

**TABLE 1:** Clinical characteristics of the study population

Variables	Healthy control (*n* = 106)	TIA patients (*n* = 189)	*p* value
Age (years)	62.75 ± 9.04	63.46 ± 8.36	0.498
Male	70	131	0.563
BMI (kg/m^2^)	25.53 ± 3.98	26.48 ± 5.09	0.096
Hypertension	65	122	0.581
Diabetes	38	73	0.637
Dyslipidemia	37	69	0.783
ABCD^2^ score	–	4.78 ± 1.59	–
Carotid stenosis degree			–
Mild (<50%)	–	12	
Moderate (50%–70%)	–	59	
Severe (≥70%)	–	118	

Abbreviations: BMI, body mass index; TIA, transient ischemic attack.

### 3.2 Overexpression of serum miR‐200b‐3p in patients with TIA

**FIGURE 1:**
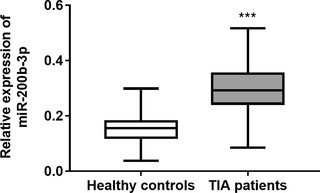
The expression of serum miR‐301a‐3p in transient ischemic attack (TIA) patients and healthy controls. The expression level of serum miR‐200b‐3p was significantly increased in TIA patients compared with healthy controls.***p < 0.001

The RT‐qPCR results are shown in Figure 1, which indicate that the expression levels of serum miR‐200b‐3p were significantly increased in TIA patients compared with healthy controls (*p* < 0.001).

### 3.3 Diagnostic value of serum miR‐200b‐3p in patients with TIA

**FIGURE 2:**
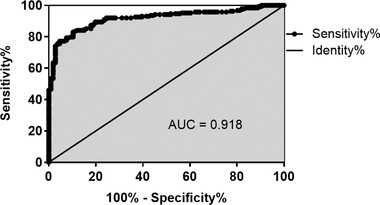
Diagnostic value of serum miR‐200b‐3p in patients with transient ischemic attack (TIA). Serum miR‐200b‐3p has the significant ability to discriminate TIA patients from healthy controls (area under the curve [AUC] = 0.918)

The ROC curve was plotted to analyze the diagnostic value of serum miR‐200b‐3p in distinguishing TIA patients from healthy controls. As shown in Figure 2, serum miR‐200b‐3p has the significant ability to discriminate TIA patients from healthy controls (area under the curve [AUC] = 0.918), indicating that serum miR‐200b‐3p had significant diagnostic value in TIA patients. At a cut‐off value of 0.219, the sensitivity was 83.07% and the specificity was 89.62%.

### 3.4 Serum miR‐200b‐3p is associated with ABCD^2^ score and ultrasound examination results in TIA patients

To facilitate the relationship analysis between miR‐200b‐3p and TIA patients’ clinical data, miR‐200b‐3p expression was divided into low (*n* = 92) and high (*n* = 97) expression groups according to the median expression value. Analysis results indicated that serum miR‐200b‐3p was significantly associated with dyslipidemia (*p* = 0.046), ABCD^2^ score (*p* < 0.001), and carotid stenosis degree on ultrasound (*p* < 0.001) in TIA patients, while association was found between miR‐200b‐3p and age, male, BMI, hypertension or diabetes (all *p* > 0.05) (Table 2).

**TABLE 2:** Association between serum miR‐200b‐3p expression level and clinical characteristics in transient ischemic attack (TIA) patients

Variables	Low miR‐200b‐3p (n = 92)	High miR‐200b‐3p (n = 97)	*p* value
Age (years)	63.00 ± 8.18	63.89 ± 8.55	0.468
Male	61	70	0.383
BMI (kg/m^2^)	26.38 ± 4.72	26.58 ± 5.44	0.780
Hypertension	56	66	0.303
Diabetes	30	43	0.098
Dyslipidemia	27	42	0.046
ABCD^2^ score	3.87 ± 1.44	4.92 ± 1.36	<0.001
Carotid stenosis degree			<0.001
Mild (<50%)	10	2	
Moderate (50%–70%)	37	22	
Severe (≥70%)	45	73	

Abbreviation: BMI, body mass index.

### 3.5 Synthetic role of miR‐200b‐3p, ABCD^2^ score, and carotid ultrasound in the prediction of CI in TIA patients

**FIGURE 3:**
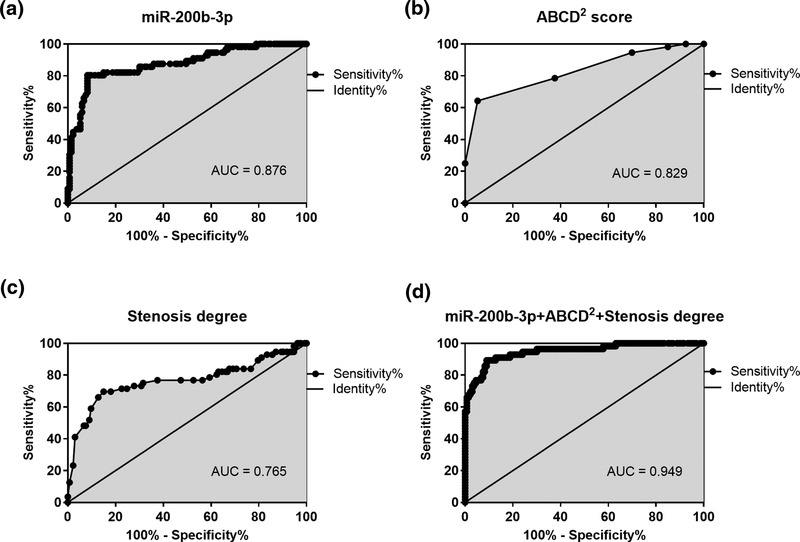
Synthetic role of miR‐200b‐3p, ABCD^2^ score, and carotid ultrasound in the prediction of cerebral infarction (CI) in transient ischemic attack (TIA) patients. (a) Serum miR‐200b‐3p had the accuracy in the prediction of CI onset in TIA patients (area under the curve [AUC] = 0.876). (b) ABCD^2^ score had the accuracy in the prediction of CI onset in TIA patients (AUC = 0.829). (c) Carotid stenosis degree on ultrasound had the accuracy in the prediction of CI onset in TIA patients (AUC = 0.765). (d) The accuracy was best for the combination of miR‐200b‐3p, ABCD^2^ score, and carotid stenosis degree on ultrasound in the prediction of CI onset in TIA patients (AUC = 0.949)

TIA is a major risk factor leading to the occurrence of CI. During the 30‐day follow‐up period, 56 TIA patients (29.6%) progressed to CI. Logistic regression analysis revealed that ABCD^2^ score (*p* = 0.011), carotid stenosis degree on ultrasound (*p* = 0.031), and serum miR‐200b‐3p (*p* = 0.006) were independently associated with CI onset in TIA patients (Table 3). Based on the results of logistic regression analysis, we separately analyzed the accuracy of miR‐200b‐3p, ABCD^2^ score, and carotid stenosis degree by ultrasound in predicting the occurrence of CI. The results of the analysis are shown in Figure 3; miR‐200b‐3p (AUC = 0.876), ABCD^2^ score (AUC = 0.829), and carotid stenosis degree on ultrasound (AUC = 0.765) all had the accuracy in the prediction of CI onset in TIA patients (Figure 3a–c). In addition, the best prediction accuracy was found when combining miR‐200b‐3p, ABCD^2^ score, and carotid stenosis degree for the risk of CI in TIA patients (Figure 3d, AUC = 0.949).

**TABLE 3:** Logistic regression analysis for cerebral infarction (CI) onset in transient ischemic attack (TIA) patients

Variables	Univariate analysis		Multiple analysis	
	OR (95% CI)	*p* value	OR (95% CI)	*p* value
Age	1.331 (0.755–1.964)	0.286	–	–
Male	1.104 (0.703–1.515)	0.379	–	–
BMI	1.472 (0.914–2.015)	0.112	–	–
Hypertension	1.356 (0.878–1.914)	0.234	–	–
Diabetes	1.287 (0.738–1.808)	0.327	–	–
Dyslipidemia	1.222 (0.763–1.759)	0.296	–	–
ABCD^2^ score	2.641 (1.562–3.723)	0.008	2.434 (1.448–3.497)	0.011
Carotid stenosis degree	2.320 (1.385–3.292)	0.027	2.079 (1.259–2.993)	0.031
miR‐200b‐3p	2.883 (1.714–3.994)	0.002	2.625 (1.638–3.635)	0.006

Abbreviations: BMI, body mass index; CI, confidence interval.

## 4 DISCUSSION

Multiple studies have confirmed that miR‐200b‐3p was aberrantly expressed in many mechanistic processes and associated with the progression of diseases. For instance, Zhou et al. found that miR‐200b‐3p was downregulated in melanoma tissues and cell lines, and promoted melanoma progression via activation of EMT (Zhou et al., 2020). Besides, decreased miR‐200b‐3p could lead to angiogenesis by enhancing endothelial ERG expression (Moh‐Moh‐Aung et al., 2020). Moreover, Zhang et al. (2021) revealed that the overexpression of miR‐200b‐3p could promote endothelial cells apoptosis under oxidative stress in atherosclerosis. In our present study, we found that the expression of serum miR‐200b‐3p was significantly increased in TIA patients compared with healthy controls. Therefore, we speculated that serum miR‐200b‐3p might be involved in TIA progression.

The occurrence of TIA caused great damage to human psychology and physiology (Miao et al., 2016; van Rooij et al., 2014), and TIA was regarded as a major risk factor for the occurrence of CI (Wang et al., 2020). Therefore, it was of great importance to make an accurate and early diagnosis of TIA patients. Many factors that could be used as diagnostic biomarkers for TIA have been confirmed in previous studies. In general, ABCD^2^ score was considered an important method for the diagnosis of TIA and the prevention of stroke (Coutts, 2017), but its accuracy in predicting CI in TIA remains limited (Wardlaw et al., 2015). Besides, Skarpengland et al. (2018) revealed that increased level of lectin‐like oxidized low‐density lipoprotein receptor‐1 in TIA was considered as a diagnostic biomarker for TIA. Moreover, serum miR‐126 expression was found to be significantly reduced after onset of TIA, and at the same time miR‐126 was significantly associated with secondary CI after onset of TIA (Lidong et al., 2021). In this study, ROC curve results indicated that serum miR‐200b‐3p has the significant ability to discriminate TIA patients from healthy controls, indicating that serum miR‐200b‐3p had significant diagnostic value in TIA patients. From the above finding, we considered that serum miR‐200b‐3p might be a diagnostic biomarker for TIA patients.

miR‐200b‐3p has been authenticated to be related to pivotal mechanistic in the pathogenesis of TIA. According to previous studies, carotid stenosis caused by carotid web lesions was a risk factor for the occurrence of TIA (Hu et al., 2019) and changes in miR‐200b‐3p expression were found in degenerative aortic stenosis patient tissues (Shi et al., 2016). Meanwhile, another study suggested a link between miR‐200b‐3p and lipid metabolism (Wu et al., 2021). Thus, it can be deduced that miR‐200b‐3p might be closely related with carotid stenosis and dyslipidemia. These speculations have been verified in the present study. Statistical analysis results of clinical characteristics indicated that serum miR‐200b‐3p was significantly associated with dyslipidemia, ABCD^2^ score, and carotid stenosis degree in TIA patients.

Various indicators had predictive roles in the occurrence of CI after TIA. ABCD^2^ score was the most instructive prediction tool for the occurrence of CI in TIA patients (Cutting et al., 2016). Carotid ultrasonography also played an important role in clinical diagnosis and follow‐up of TIA patients (Kilic Coban et al., 2020). Additionally, other miRNAs also had potential as predictive biomarkers in predicting CI onset after TIA, such as miR‐126 (Lidong et al., 2021). As far as miR‐200b‐3p was concerned, in terms of cerebrovascular disease, articles from gene polymorphism studies indicated that miR‐200b‐3p was significantly associated with stroke susceptibility and mortality (Kim et al., 2016). CI was a kind of an ischemic stroke (Gao & Zhang, 2017). Therefore, this study analyzed the ability of above indicators to predict the occurrence of CI. First, logistic regression analysis revealed that ABCD^2^ score, carotid stenosis degree on ultrasound, and serum miR‐200b‐3p were independently associated with CI onset in TIA patients. Second, miR‐200b‐3p, ABCD^2^ score, and carotid stenosis degree had accuracy in the prediction of CI onset in TIA patients, and the accuracy of the combination of miR‐200b‐3p, ABCD^2^ score, and carotid stenosis degree was best in the prediction of CI onset in TIA patients. The above study has proved that the synthetic role of miR‐200b‐3p, ABCD^2^ score, and carotid stenosis degree might be a good method to screen TIA patients at high risk of CI.

The specific mechanism or signaling pathways by which serum miR‐200b‐3p participated in the progression of TIA has not been explored in depth in our study, which was the limitation of this article as well as the direction of our next research. Wu et al. (2021) has reported that the knockdown of miR‐200b‐3p alleviated lipid accumulation and promoted cholesterol efflux, which were two characteristics in the pathogenesis of atherosclerosis, by targeting ABCA^1^. Another study found that miR‐200b‐3p was involved in the regulation of angiogenesis by directly regulating the vascular endothelial growth factor (VEGF) signaling (Choi et al., 2011). Additionally, in atherosclerosis, miR‐200b‐3p has been found to enhance endothelial cell injury through targeting HDAC4 (Zhang et al., 2021). These previous studies indicated that miR‐200b‐3p might be involved in the development of CI by affecting lipid accumulation and angiogenesis, but the mechanisms and related targets and pathways need to be verified and explored in future studies.

Taken together, our study indicated that serum miR‐200b‐3p was increased in TIA patients compared with healthy controls and had significant diagnostic value in TIA patients. Furthermore, miR‐200b‐3p was associated with ABCD^2^ score and carotid stenosis degree in TIA patients, and the combination of the above three indicators showed the considerable predictive accuracy for CI onset in TIA patients. Therefore, we speculated that the joint detection of miR‐200b‐3p, ABCD^2^ score, and carotid stenosis degree may help to improve the predictive strategy of CI in TIA patients.

## AUTHOR CONTRIBUTIONS

Jiwei Zheng carried out the research design and conception; Zhirong Wang and Xiaoguang Huo analyzed and interpreted the data; Zhirong Wang and Na Li performed the examination of sample; Jiwei Zheng and Xiaomeng Zhang contributed essential reagents or tools. All authors have written and revised the manuscript. All authors have read and approved the final manuscript. 

## ETHICS APPROVAL AND CONSENT TO PARTICIPATE

The experimental procedures were approved and in accordance with the guidelines of the Ethics Committee of Huantai People's Hospital. This study complies with the Declaration of Helsinki. A written and signed informed consent was obtained from each patient.

## CONSENT FOR PUBLICATION

A written informed consent for publication was obtained from each participant.

## CONFLICT OF INTEREST

The authors declare no conflict of interest.

## Data Availability

The data used and analyzed can be obtained from the corresponding author under a reasonable request.

## References

[brb32518-bib-0001] Choi, Y. C. , Yoon, S. , Jeong, Y. , Yoon, J. , & Baek, K. (2011). Regulation of vascular endothelial growth factor signaling by miR‐200b. Molecules and Cells, 32(1), 77–82. 10.1007/s10059-011-1042-2 21544626 PMC3887663

[brb32518-bib-0002] Coutts, S. B. (2017). Diagnosis and management of transient ischemic attack. Continuum (Society for Social Work Administrators in Health Care), 23(1), 82–92.

[brb32518-bib-0003] Cutting, S. , Regan, E. , Lee, V. H. , & Prabhakaran, S. (2016). High ABCD2 scores and in‐hospital interventions following transient ischemic attack. Cerebrovascular Diseases Extra, 6(3), 76–83. 10.1159/000450692 27721312 PMC5091225

[brb32518-bib-0004] Easton, J. D. , Saver, J. L. , Albers, G. W. , Alberts, M. J. , Chaturvedi, S. , Feldmann, E. , Hatsukami, T. S. , Higashida, R. T. , Johnston, S. C. , Kidwell, C. S. , Lutsep, H. L. , Miller, E. , & Sacco, R. L. , & American Heart Association ; American Stroke Association Stroke Council ; Council on Cardiovascular Surgery and Anesthesia ; Council on Cardiovascular Radiology and Intervention; Council on Cardiovascular Nursing; Interdisciplinary Council on Peripheral Vascular Disease . (2009). Definition and evaluation of transient ischemic attack: A scientific statement for healthcare professionals from the American Heart Association/American Stroke Association Stroke Council; Council on Cardiovascular Surgery and Anesthesia; Council on Cardiovascular Radiology and Intervention; Council on Cardiovascular Nursing; and the Interdisciplinary Council on Peripheral Vascular Disease. The American Academy of Neurology affirms the value of this statement as an educational tool for neurologists. Stroke; A Journal of Cerebral Circulation, 40(6), 2276–2293. 10.1161/STROKEAHA.108.192218 19423857

[brb32518-bib-0005] Eckstein, H. H. , Kuhnl, A. , Dorfler, A. , Kopp, I. B. , Lawall, H. , & Ringleb, P. A. , & Multidisciplinary German‐Austrian guideline based on evidence and consensus . (2013). The diagnosis, treatment and follow‐up of extracranial carotid stenosis. Deutsches Ärzteblatt International, 110(27‐28), 468–476. 23964303 10.3238/arztebl.2013.0468 PMC3722642

[brb32518-bib-0006] Gao, J. , & Zhang, H. J. (2017). Effects of chin tuck against resistance exercise versus Shaker exercise on dysphagia and psychological state after cerebral infarction. European Journal of Physical and Rehabilitation Medicine, 53(3), 426–432. 10.23736/S1973-9087.16.04346-X 27830923

[brb32518-bib-0007] Giles, M. F. , Albers, G. W. , Amarenco, P. , Arsava, E. M. , Asimos, A. W. , Ay, H. , Calvet, D. , Coutts, S. B. , Cucchiara, B. L. , Demchuk, A. M. , Johnston, S. C. , Kelly, P. J. , Kim, A. S. , Labreuche, J. , Lavallee, P. C. , Mas, J.‐L. , Merwick, A. , Olivot, J. M. , Purroy, F. , … Rothwell, P. M. (2017). Early stroke risk and ABCD2 score performance in tissue‐ vs time‐defined TIA: A multicenter study. Neurology, 77(13), 1222–1228. 10.1212/WNL.0b013e3182309f91 PMC3179650 21865578

[brb32518-bib-0008] Hu, H. , Zhang, X. , Zhao, J. , Li, Y. , & Zhao, Y. (2019). Transient ischemic attack and carotid web. AJNR American Journal of Neuroradiology, 40(2), 313–318. 10.3174/ajnr.A5946 30655258 PMC7028627

[brb32518-bib-0009] Johnston, S. C. , Rothwell, P. M. , Nguyen‐Huynh, M. N. , Giles, M. F. , Elkins, J. S. , Bernstein, A. L. , & Sidney, S. (2007). Validation and refinement of scores to predict very early stroke risk after transient ischaemic attack. Lancet, 369(9558), 283–292. 10.1016/S0140-6736(07)60150-0 17258668

[brb32518-bib-0010] Kernan, W. N. , Ovbiagele, B. , Black, H. R. , Bravata, D. M. , Chimowitz, M. I. , Ezekowitz, M. D. , Fang, M. C. , Fisher, M. , Furie, K. L. , Heck, D. V. , Johnston, S. C. C. , Kasner, S. E. , Kittner, S. J. , Mitchell, P. H. , Rich, M. W. , Richardson, D. , Schwamm, L. H. , & Wilson, J. A. , & American Heart Association Stroke Council, Council on Cardiovascular and Stroke Nursing , Council on Clinical Cardiology, and Council on Peripheral Vascular Disease . (2014). Guidelines for the prevention of stroke in patients with stroke and transient ischemic attack: A guideline for healthcare professionals from the American Heart Association/American Stroke Association. Stroke; A Journal of Cerebral Circulation, 45(7), 2160–1236. 10.1161/STR.0000000000000024 24788967

[brb32518-bib-0011] Khorvash, F. , Hemasian, H. , Shahabi, S. , Shahzamani, A. , Sheikhbahaei, E. , & Chitsaz, A. (2018). Predicting long‐term cardiovascular events after transient ischemic attacks: Carotid artery intima‐media thickness or ABCD2 score or both? International Journal of Preventive Medicine, 9, 102. 30598740 10.4103/ijpvm.IJPVM_415_17 PMC6259433

[brb32518-bib-0012] Kilic Coban, E. , Senadim, S. , Yilmaz, A. , Kucukoglu, H. , Koksal, A. , Atakli, D. , & Soysal, A. (2020). The review of transient ischemic attack patients: An experience of a clinic about diagnosis and follow‐up. Sisli Etfal Hastanesi Tip Bulteni, 54(1), 83–87. 32377139 10.14744/SEMB.2018.20438 PMC7192245

[brb32518-bib-0013] Kim, J. M. , Jung, K. H. , Chu, K. , Lee, S. T. , Ban, J. , Moon, J. , Kim, M. , Lee, S. K. , & Roh, J.‐K. (2015). Atherosclerosis‐related circulating microRNAs as a predictor of stroke recurrence. Translational Stroke Research, 6(3), 191–197. 10.1007/s12975-015-0390-1 25697638

[brb32518-bib-0014] Kim, J. , Choi, G. H. , Ko, K. H. , Kim, J. O. , Oh, S. H. , Park, Y. S. , Kim, O. J. , & Kim, N. K. (2016). Association of the single nucleotide polymorphisms in microRNAs 130b, 200b, and 495 with ischemic stroke susceptibility and post‐stroke mortality. PLoS One, 11(9), e0162519. 10.1371/journal.pone.0162519 27603512 PMC5014326

[brb32518-bib-0015] Liang, Z. , Chi, Y. J. , Lin, G. Q. , Luo, S. H. , Jiang, Q. Y. , & Chen, Y. K. (2018). MiRNA‐26a promotes angiogenesis in a rat model of cerebral infarction via PI3K/AKT and MAPK/ERK pathway. European Review for Medical and Pharmacological Sciences, 22(11), 3485–3492. 29917203 10.26355/eurrev_201806_15175

[brb32518-bib-0016] Lidong, D. , Zhanghong, X. , Huawu, M. , Xiaofang, H. , Junhua, G. , Kaifu, K. , & Jue, C. (2021). Ischemia modified albumin and mir‐126 play important role in diagnosis of posterior circulation transient ischemic attack and prediction of secondary cerebral infarction. Neurology India, 69(1), 75–80. 10.4103/0028-3886.310100 33642274

[brb32518-bib-0017] Longstreth, W. T. Jr. , Gasca, N. C. , Gottesman, R. F. , Pearce, J. B. , & Sacco, R. L. (2018). Adjudication of transient ischemic attack and stroke in the multi‐ethnic study of atherosclerosis. Neuroepidemiology, 50(1‐2), 23–28. 10.1159/000486174 29324452 PMC5860960

[brb32518-bib-0018] Miao, M. S. , Guo, L. , Li, R. Q. , & Zhang, X. L. (2016). Radix Ilicis Pubescentis total flavonoids ameliorates neuronal damage and reduces lesion extent in a mouse model of transient ischemic attack. Neural Regeneration Research, 11(3), 441–446. 10.4103/1673-5374.179056 27127483 PMC4829009

[brb32518-bib-0019] Moh‐Moh‐Aung, A. , Fujisawa, M. , Ito, S. , Katayama, H. , Ohara, T. , Ota, Y. , Yoshimura, T. , & Matsukawa, A. (2020). Decreased miR‐200b‐3p in cancer cells leads to angiogenesis in HCC by enhancing endothelial ERG expression. Science Reports, 10(1), 10418. 10.1038/s41598-020-67425-4 PMC7320004 32591615

[brb32518-bib-0020] Paciaroni, M. , Eliasziw, M. , Kappelle, L. J. , Finan, J. W. , Ferguson, G. G. , & Barnett, H. J. (1999). Medical complications associated with carotid endarterectomy. North American Symptomatic Carotid Endarterectomy Trial (NASCET). Stroke; A Journal of Cerebral Circulation, 30(9), 1759–1763. 10.1161/01.STR.30.9.1759 10471420

[brb32518-bib-0021] Rafailidis, V. , Charitanti, A. , Tegos, T. , Destanis, E. , & Chryssogonidis, I. (2017). Contrast‐enhanced ultrasound of the carotid system: A review of the current literature. Journal of Ultrasound, 20(2), 97–109. 10.1007/s40477-017-0239-4 28592999 PMC5440332

[brb32518-bib-0022] Shi, J. , Liu, H. , Wang, H. , & Kong, X. (2016). MicroRNA expression signature in degenerative aortic stenosis. BioMed Research International, 2016. 10.1155/2016/4682172 PMC4989063 27579316

[brb32518-bib-0023] Skarpengland, T. , Skjelland, M. , Kong, X. Y. , Skagen, K. , Holm, S. , Otterdal, K. , Dahl, C. P. , Krohg‐Sørensen, K. , Sagen, E. L. , Bjerkeli, V. , Aamodt, A. H. , Abbas, A. , Gregersen, I. , Aukrust, P. , Halvorsen, B. , & Dahl, T. B. (2018). Increased levels of lectin‐like oxidized low‐density lipoprotein receptor‐1 in ischemic stroke and transient ischemic attack. Journal of the American Heart Association, 7(2), e006479. 10.1161/JAHA.117.006479 29330254 PMC5850141

[brb32518-bib-0024] van Rooij, F. G. , Schaapsmeerders, P. , Maaijwee, N. A. , van Duijnhoven, D. A. , de Leeuw, F. E. , Kessels, R. P. , & van Dijk, E. J. (2014). Persistent cognitive impairment after transient ischemic attack. Stroke; A Journal of Cerebral Circulation, 45(8), 2270–2274. 10.1161/STROKEAHA.114.005205 25070959

[brb32518-bib-0025] Wang, J. , Zhang, P. , & Tang, Z. (2020). Animal models of transient ischemic attack: A review. Acta Neurologica Belgica, 120(2), 267–275. 10.1007/s13760-020-01295-5 32048230 PMC7083805

[brb32518-bib-0026] Wardlaw, J. M. , Brazzelli, M. , Chappell, F. M. , Miranda, H. , Shuler, K. , Sandercock, P. A. , & Dennis, M. S. (2015). ABCD2 score and secondary stroke prevention: Meta‐analysis and effect per 1,000 patients triaged. Neurology, 85(4), 373–380. 10.1212/WNL.0000000000001780 26136519 PMC4520819

[brb32518-bib-0027] Wu, Y. T. , Li, J. B. , Lin, H. Q. , Zhang, G. X. , Hong, C. M. , Li, M. , Guo, Z.‐ J. , & Yang, Y.‐ B. (2021). Inhibition of miR‐200b‐3p alleviates lipid accumulation and promotes cholesterol efflux by targeting ABCA1 in macrophage‐derived foam cells. Experimental and Therapeutic Medicine, 22(2), 831. 10.3892/etm.2021.10263 34149877 PMC8200800

[brb32518-bib-0028] Zhang, C. , Zang, Y. , Hu, L. , Song, Q. , Zhao, W. , Zhang, C. , Liu, H. , & Gu, F. (2020). Study on the risk prediction for cerebral infarction after transient ischemic attack: A STROBE compliant study. Medicine, 99(11), e19460. 10.1097/MD.0000000000019460 32176078 PMC7220422

[brb32518-bib-0029] Zhang, F. , Cheng, N. , Du, J. , Zhang, H. , & Zhang, C. (2021). MicroRNA‐200b‐3p promotes endothelial cell apoptosis by targeting HDAC4 in atherosclerosis. BMC Cardiovascular Disorders, 21(1), 172. 10.1186/s12872-021-01980-0 33845782 PMC8042726

[brb32518-bib-0030] Zhou, W. J. , Wang, H. Y. , Zhang, J. , Dai, H. Y. , Yao, Z. X. , Zheng, Z. , Meng‐Yan, S. , & Wu, K. (2020). NEAT1/miR‐200b‐3p/SMAD2 axis promotes progression of melanoma. Aging, 12(22), 22759–22775. 33202380 10.18632/aging.103909 PMC7746346

